# Assessment of selected heavy metals and enzyme activity in soils within the zone of influence of various tree species

**DOI:** 10.1038/s41598-020-69545-3

**Published:** 2020-08-21

**Authors:** Agata Bartkowiak, Joanna Lemanowicz, Robert Lamparski

**Affiliations:** 1 Department Biogeochemistry and Soil Science, Faculty of Agriculture and Biotechnology, UTP University of Science and Technology in Bydgoszcz, 6 Bernardyńska St., 85-029 Bydgoszcz, Poland; 2 Department of Entomology and Molecular Phytopathology, Faculty of Agriculture and Biotechnology, UTP University of Science and Technology in Bydgoszcz, 7 Prof. S. Kaliskiego St., 85-796 Bydgoszcz, Poland

**Keywords:** Biogeochemistry, Ecology, Environmental sciences

## Abstract

The study aimed to evaluate the total content and bioavailable forms of Zn, Cu, Pb and Ni and enzymatic activity (nitro reductase and peroxidases) in the mineral levels of surface soils within the zone of influence of various tree species. The conducted variance analysis confirmed the significant impact of the studied tree habitats on the total content and bioavailable forms of metals and on enzymatic activity. The total content of analysed metals were low and in no case exceeded the possible concentrations. The high bioavailability (*AF* %) values calculated for habitats of different species compositions (of 53.78% for Zn, 76.82% for Cu, 60.81% for Pb and 44.72% for Ni) may pose a risk of accumulation of these metals in plants. A significant correlation was found between nitrate reduction activity and Pb content (*r* = 0.510) and Cu (*r* = 0.678). Principal component analysis allowed two principal components to be distinguished (*PC1* and *PC2*) that accounted for 60.95% of the total change in variance.

## Introduction

The living environment of human beings is predominantly urban. Urban agglomeration parks are a biogeochemical barrier in an urban landscape and fulfil positive functions by reducing soil erosion, limit the spread of chemical compounds, control matter cycling and protect the accumulation of toxic chemicals. The soil and air of these areas contain elevated concentrations of heavy metals. However, the state of the soil environment in urban areas is random. It depends on the climatic conditions, topography, development, industrialisation of the area, and above all else the intensity of road traffic^1,2,3,4^. The heavy-metal pollution of the environment is a global problem, because increased concentrations in the ecosystem have become a serious ecological problem due to the harmful effect they have on plant and animal organisms^2,3,5^. Estimating trace-metal bioavailability should be the main aspect on which to base assessment of plant potential to activate and accumulate metals from soil^6,7^. Of particular use in cleaning up the environment are plants, which not only produce oxygen, but also take up and accumulate pollutants from the soil and air, primarily in leaves and roots. The spatial structure of urban vegetation usually reflects the urban layout of the city, but the quality of greenery in the city centre is mainly determined by anthropogenic factors. Vegetation in cities improves inhabitants quality of life. These benefits stem primarily from the positive impact that plants have on human well-being. Urban trees and shrubs purify the air by capturing particulate matter, including a significant amount of heavy metals. The species composition of trees has a diverse impact on the physical and chemical properties of soil. They primarily affect soil properties by supplying organic matter of differing quantity and quality^8^. This results in an increase in the abundance and richness of soil mesofauna, changes in nitrogen and carbon content, and changes in soil pH, which are accompanied by an increase or decrease in the mobility of some metals^9,10,11,12,13^. The species composition of a tree stand can determine the diversity of microorganisms and their enzymatic activity; trees also determine the spatial distribution and quantities of soil enzymes^14^.

Soil enzymes, as natural catalysts of many soil processes related to the decomposition of organic matter, participate in the processes of releasing mineral substances and supplying them to plant organisms. Enzymatic activity is an early indicator of changes in the intensity of biological processes and the level of soil degradation and usually correlates with physical and chemical soil properties^15,16^. Enzymes may play an important role in the phytoremediation process after plant death. These include, enzymes e.g. from the class of oxidoreductases: dehydrogenases, nitrate reductase, catalase and peroxidases with significant catalytic activity in relation to many organic contaminants. In urban conditions, the accumulation of unfavourable soil properties (xerisation, salinity, toxication) contributes to changing the course of physiological and biochemical plant processes, which in turn interferes with morphology and reduces their decorative qualities. Nitrate reductase is an enzyme participating in the process of denitrification. This enzyme reduces nitrate to nitrite. Next, the formed NO_2_ ions are reduced with the participation of nitrite reductase to N_2_O. Peroxidase activity in the soil is poorly studied. Peroxidases resent in natural soil originate from microorganisms, plants, or other organisms. According to Bach^17^. these enzymes participate the biogeochemical processes of lignin degradation, oxidation of toxic substances carbon mineralization and sequestration.

The aim of this work was to assess the total and bioavailable forms of selected heavy metals (Zn, Cu, Pb and Ni) and enzymatic activity (nitro reductase and peroxidases) in the mineral levels of surface soils within the zone of influence of various tree species. The following research project is a continuation of monitoring studies within the Forest Park of Culture and Recreation in Bydgoszcz. These studies will help determine the right selection of trees in urban parks, which will positively affect soil biodiversity and its protection.

## Material and methods

### Location of soil sampling

**Figure 1:**
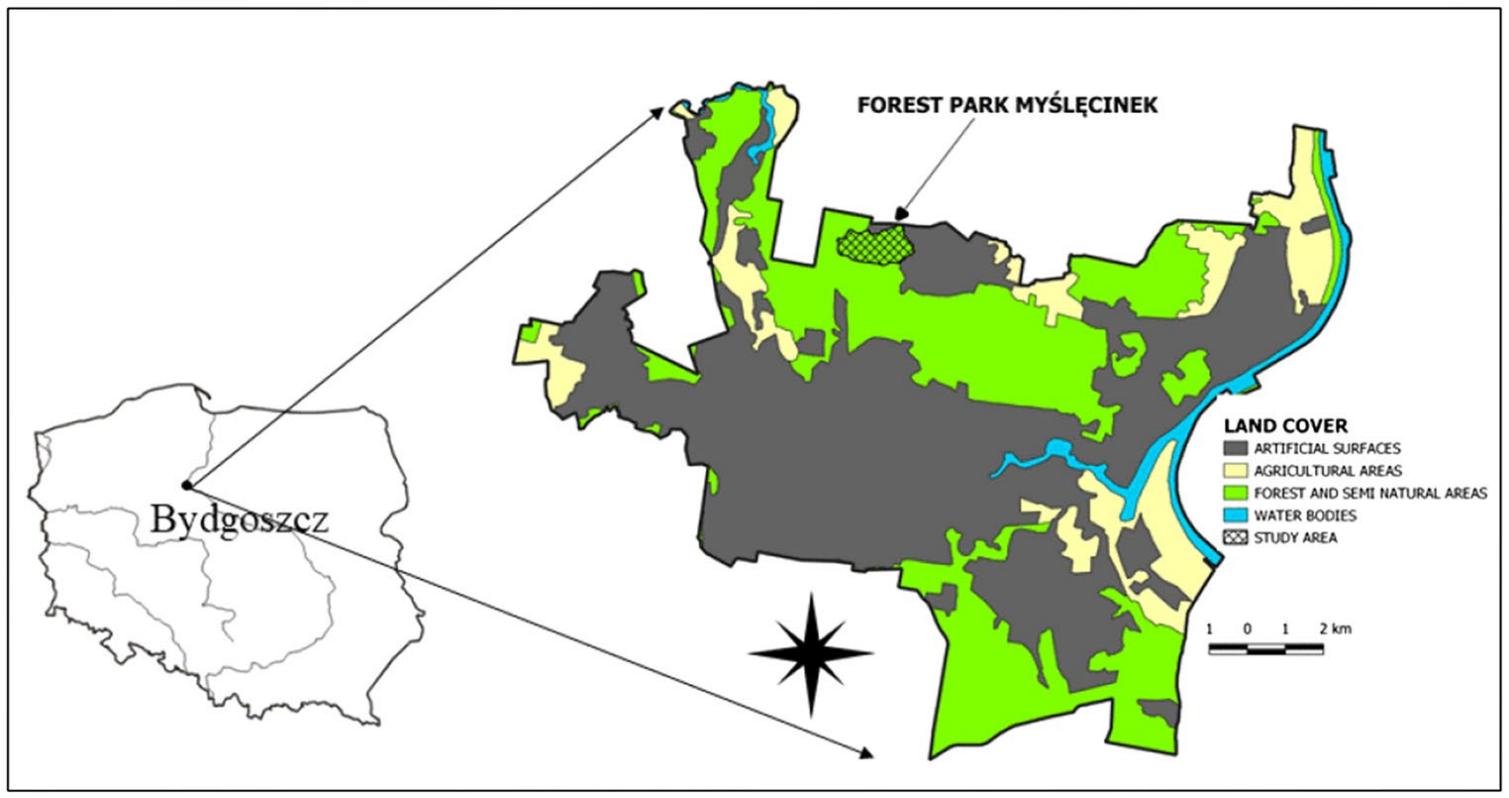
Localization study area (map was generated based to CORINE Land Cover 2018 database (https://www.clc.gios.gov.pl) using free and open source software QGIS 3.4. The Corine Land Cover 2018 project in Poland was implemented by the Institute of Geodesy and Cartography and financed by the European Union. The results of the project were obtained from the website of the Chief Inspectorate for Environmental Protection).

The research was carried out within the Forest Park of Culture and Recreation in Bydgoszcz (53° 09′ 41, 07′ N, 18° 00′ 50, 29′ E), in the Kuyavian Pomeranian Province, central Poland) (Fig. 1). Bydgoszcz is one of Poland’s largest cities, ranking 8th by population, which in 2017 was around 400,000, and 11th by area (176 km^2^). It is a major centre of industry, trade and logistics, as well as being a road, rail and inland waterways intersection. Myślęcinek is the largest city park in Poland, with 830 ha, and is located in the north of Bydgoszcz, 5 km from the city centre; it covers 4.7% of the city. The Forest Park of Culture and Recreation is a large recreational and leisure area combining the natural beauty with infrastructure for active recreation, relaxation and sport. The park has a varied relief (52–107 m a.s.l.) and a variety of vegetation: from moist and wetlands to dry environments. More than half of the park is forested. The forest stand consists mainly of pine, mixed and broadleaf forests. In the north of the park there is a 60-ha botanical garden from which study samples were taken. For the research, nine areas of different species habitats were selected: (1) saucer magnolia (*Magnolia* × *soulangeana*); (2) European hornbeam (*Carpinus betulus* L.); (3) common hawthorn (*Crataegus monogyna* Jacq.); (4) northern white-cedar (*Thuja occidentalis L.*); (5) European oak (*Quercus robur* L.); (6) common hazel (*Corylus avellana* L.); (7) katsura (*Cercidiphyllum japonicum* Siebold & Zucc.); (8) silver birch (*Betula pendula* Roth); (9) black alder (*Alnus glutinosa* Gaertn.).

### Reagents

All chemicals were of analytical grade, as very low concentrations of trace metals were required for this study. Double-deionised water from a Millipore system was used to prepare solutions and dilutions.

### Soil analysis

#### Physicochemical parameters of the soil

Soil samples were collected in spring (May 2018) from surface mineral levels from depths of 0–30 cm. In total, 45 samples were analysed. Each sample was a mixture of five subsamples taken at random places within each examined habitat.

In air-dried disturbed soil samples sieved through a ø 2-mm mesh, selected physicochemical properties were determined: pH in H_2_O solutions and in 1 M KCl (PN-ISO 10390)^18^; total organic carbon (TOC) and total nitrogen (N) using a Skalar TOC Primacs analyser; and granulometric composition by laser diffraction method using a Masterssizer MS 2000 analyser. The total content of zinc, copper, lead and nickel was determined after mineralisation in a mixture of HF + HClO_4_ acids according to the method of Crock and Severson^19^. Obtained total contents of analysed heavy metals were compared with EPA^20^ standards and of the Regulation Minister of Environment^21^. Bioavailable forms were extracted with 1 M HCl by Rinkins’ method. The content of heavy metals in the extracts obtained by the aforementioned procedures was determined by atomic absorption spectroscopy using a Philips PU 9100X spectrometer. To verify the accuracy of the results, the analysis of the certified material Loam Soil No. ERM-CC141 as well as the so-called zero tests were made, which were exposed to the identical analytic procedure as the soil samples. Good compatybility between the certified and determined values was obtained. The limit of determination for all analysed metals were 0.2 mg kg^−1^.

The results facilitated a calculation of the availability factor (*AF*) as suggested by Obrador et al.^22^.

It is expressed as follows:

1 $$AF=\left(\frac{Available \;content}{Total\; content}\right) \times 100$$

where *AF* is the availability factor (%).

#### Enzymes analysis

Moist test soils were sieved (2-mm mesh) and stored in plastic boxes at 4 °C for a period of not less than 2 days to stabilise microbial activity, and analysed for activity of nitrate reductase (NR) [EC 1.6.6.1] and peroxidases (PER) within 1 week. The activity of selected oxidoreductases enzymes—nitrate reductase activity (NR) was determined using KNO_3_ as a substrate and 2,4-dinitrophenol as inhibitor of nitrite reductase according to Kandeler method^21^. Nitrite released as a result of incubation was extracted with potassium chloride solution and determined colorimetrically at λ = 520 nm. Analyses of peroxidases activity [EC 1.11.1.7] were carried out using the Bartha and Bordeleau method^23^ by measuring the amount of purpurogallin (PPG) formed as a result of the oxidation of pyrogallol in the presence of H_2_O_2_. The absorbance of the solution was measured colorimetrically at λ = 460 nm using a spectrophotometer.

#### Data analysis

In order to investigate the impact of plant habitat on TOC, N, total content and bioavailable forms of the analysed heavy metals and the activity of selected enzymes, a single-factor analysis of variance (ANOVA) was performed. Differentiation of means for objects was determined by identifying homogeneous groups based on the LSD test with a significance level of α = 0.05. The contents of the investigated heavy metals, TOC and N, and granulometric composition, soil reaction and enzymatic activity were evaluated using principal component analysis (PCA). In addition, Pearson linear correlation coefficients, regression equation and coefficient of determination (*R*^*2*^) were determined between all analysed variables using the Statistica 13.0 computer program. All analytical measurements were performed with three replications. Arithmetic mean values are shown in tables ± standard deviation.

## Results and discussion

### Basic soil properties

The granulometric composition analysis, revealed that the analysed soil samples had a similar grain size, containing 44.37 to 67.18% sand fraction, 29.85 to 50.85% silt fraction and 2.56 to 6.23% clay fraction. The dominant granulometric group was sandy loam (USDA^24^), which was recorded in eight of the tested plant habitats. Only the hornbeam habitat was characterised by the content of granulometric fractions corresponding to loamy silt (Table 1). The analysed soil samples had a neutral reaction. The pH values corresponding to active acidity ranged from 6.75 to 8.1, and exchangeable acidity from pH 6.2 to 7.32 pH. Organic carbon content varied depending on the studied habitat, ranging from 18.8 to 46.04 g kg^−1^. The highest values of the discussed parameter were recorded at site 9 (habitats black alder) and the lowest ones at site 8 (habitats silver birch). The main source of TOC in the samples was the gradual accumulation of matter from fallen tree leaves and dead undergrowth.

**Table 1:** Selected soil physicochemical properties.

Trees	pH		Fraction%			TOC	N
	H_2_O	KCl	Sand	Silt	Clay	g kg^−1^	
1	8.10 ± 0.07	7.32 ± 0.11	67.18 ± 1.02	29.85 ± 0.95	2.97 ± 0.05	19.25^fg^ ± 0.17	1.21 ± 0.11
2	7.60 ± 0.11	6.95 ± 0.20	44.37 ± 1.25	50.85 ± 0.947	4.78 ± 0.12	25.05^e^ ± 0.58	1.73 ± 0.15
3	7.32 ± 0.21	6.79 ± 0.16	60.40 ± 1.42	37.04 ± 1.0	2.56 ± 0.14	36.85^b^ ± 0.74	3.07 ± 0.12
4	7.33 ± 0.12	6.90 ± 0.37	59.94 ± 1.22	37.24 ± 1.17	2.82 ± 0.21	33.68^c^ ± 0.12	2.51 ± 0.12
5	7.16 ± 0.30	6.35 ± 0.17	58.77 ± 1.74	38.11 ± 1.18	3.11 ± 0.09	31.87^d^ ± 0.32	2.50 ± 0.09
6	7.68 ± 0.09	7.06 ± 0.18	60.63 ± 1.42	36.36 ± 1.09	3.01 ± 0.08	20.75^f^ ± 0.18	1.46 ± 0.08
7	7.51 ± 0.10	7.16 ± 0.14	57.81 ± 1.72	39.16 ± 1.25	3.03 ± 0.1	24.38^e^ ± 0.23	2.04 ± 0.10
8	6.75 ± 0.10	6.20 ± 0.12	45.27 ± 1.64	48.50 ± 2.02	6.23 ± 0.212	18.80^g^ ± 0.13	1.56 ± 0.07
9	7.58 ± 0.14	7.08 ± 0.18	60.90 ± 1.44	36.32 ± 1.42	2.78 ± 0.14	46.04^a^ ± 0.81	3.06 ± 0.14

(1) Saucer magnolia, (2) European hornbeam, (3) common hawthorn, (4) northern white-cedar, (5) European oak, (6) common hazel, (7) katsura, (8) silver birch, (9) black alder.

± standard deviation.

### Heavy metal content and its relation to other soil parameters

The total content of analysed metals varied by sampling site. This was also confirmed by analysis of variance (Table 2). Total zinc content ranged from 24.75 to 51.50 mg kg^−1^, copper from 6.60 to 10.08 mg kg^−1^, lead from 21.60 to 39.10 mg kg^−1^ and nickel from 6.00 to 70.60 mg kg^−1^. The lowest values of all analysed metals were recorded at site 1, where the saucer magnolia trees were growing. The highest values were recorded at the oak site for zinc and lead, the alder site for copper, and hornbeam for nickel. The obtained values were low and in no case exceeded the permissible concentrations for areas designated for transport routes specified by the U.S. Environmental Protection Agency^20^ and Regulation Polish Minister for the Environment^21^ on methods for assessing pollution of the earth's surface. Such a distribution of contents may suggest that heavy metals have been stopped by vegetation acting as a protective barrier against them. The content of heavy metals in the soil largely correlates with distance from roads, traffic density, terrain, and land use^26,27^. The best protection is afforded by multi-layered vegetation. The effectiveness of isolation by tall greenery increases with the height, density and width of the insulating system^27,28^. A significant effect on the content of trace elements in the analysed soils was the pH of soils, which affects the mobility of metals, and thus also for the availability of plants^29^. Under strongly acidic conditions, the concentration of mobile forms of heavy metals available for plants in the soil solution increases, thereby increasing their accumulation in plants. This contributes greatly to reducing the actual content of the trace elements in the analysed soils. This was confirmed by the highly negative relationships that total zinc and copper have with exchangeable acidity, which were respectively *r* = -0.646; *p* = 0.0602 for zinc and *r* = -0,682; *p* = 0.0432 for copper (Table 3). The interaction of heavy metals in the soil with some of its properties was also assessed using the coefficient of determination (*R*^*2*^) and the regression equation. Based on the calculated coefficient of determination, it was found that potential acidity has a 41.7% and 46.4% influence on total contents of Zn and Cu, respectively. In no case did this impact exceed 50% (non-satisfactory fit). The linear regression equation shows that the a 1-pH increase in soil reaction resulted in a reduction of 13.65 mg kg^−1^ in total zinc content and 2.34 mg∙kg^−1^ in copper. Calculated significantly positive correlation coefficients between the total content of lead and zinc (r = 0.762; p = 0.017) and copper (r = 0.587; p = 0.097) as well as the total content of nickel and lead (r = 0.500, p = 0.177) indicate similar sources of origin of the analysed metals. The linear regression equations show that an increase in lead content contributed to varying degrees to increasing the concentration of zinc, copper and nickel in the studied soil.

**Table 2:** Total content and available forms (extracted with 1 M HCl) of heavy metals in the soil samples.

Trees	Total content forms				Available forms			
	mg kg^−1^							
	Zn	Cu	Pb	Ni	Zn	Cu	Pb	Ni
1	24.75^i^ ± 0.09	6.60^f^ ± 0.01	21.60^g^ ± 0.49	6.00^g^ ± 0.14	10.63^bc^ ± 0.23	5.07^ab^ ± 0.16	11.19^c^ ± 0.18	2.68^c^ ± 0.18
2	39.13^c^ ± 0.12	8.65^c^ ± 0.04	35.40^b^ ± 0.14	70.60^a^ ± 0.49	13.20^bc^ ± 6.70	5.64^ab^ ± 1.12	17.44^b^ ± 6.27	3.34^bc^ ± 0.75
3	33.15^g^ ± 0.08	8.18^d^ ± 0.13	26.23^e^ ± 0.0	8.50^e^ ± 0.04	13.23^bc^ ± 7.64	4.25^b^ ± 2.46	12.04^c^ ± 6.96	2.41^c^ ± 0.87
4	46.25^b^ ± 0.12	7.55^e^ ± 0.03	27.95^d^ ± 0.05	7.48^f^ ± 0.03	23.77^ab^ ± 1.65	3.87^b^ ± 0.09	15.87^bc^ ± 1.17	2.92^c^ ± 0.16
5	51.50^a^ ± 0.08	9.50^b^ ± 0.01	39.10^a^ ± 0.14	9.33^d^ ± 0.07	27.12^a^ ± 0.66	5.49^ab^ ± 0.32	22.06^a^ ± 0.12	3.58^bc^ ± 0.11
6	37.75^e^ ± 0.07	7.93^d^ ± 0.05	26.80^de^ ± 0.64	7.85^ef^ ± 0.05	20.30^ab^ ± 0.15	4.47^b^ ± 0.15	16.11^bc^ ± 1.52	3.08^bc^ ± 0.19
7	32.58^h^ ± 0.03	6.85^f^ ± 0.04	23.50^f^ ± 0.49	7.15^f^ ± 0.11	13.43^bc^ ± 0.82	4.95^ab^ ± 0.46	13.42^c^ ± 0.18	2.94^c^ ± 0.12
8	38.58^d^ ± 0.04	9.93^a^ ± 0.05	25.55^e^ ± 0.40	14.05^c^ ± 0.04	4.68^c^ ± 0.35	3.60^b^ ± 0.41	6.36^cd^ ± 0.35	3.71^bc^ ± 0.42
9	34.48^f^ ± 0.05	10.08^a^ ± 0.03	30.18^c^ ± 0.02	48.63^b^ ± 0.12	15.71^b^ ± 0.26	5.73^a^ ± 0.66	18.35^b^ ± 0.61	4.44^a^ ± 0.17

(1) Saucer magnolia, (2) European hornbeam, (3) common hawthorn, (4) northern white-cedar, (5) European oak, (6) common hazel, (7) katsura, (8) silver birch, (9) black alder.

± standard deviation.

**Table 3:** Results of analysis of regression and correlation.

Variable		Equation of linear regression	*r*	*R*^2^	P
Dependent	Independent				
Total Zn	pH KCl	y = 131.318–13.650x	− 0.646	0.417	0.0602
Total Cu	pH KCl	y = 24.4729–2.346x	− 0.682	0.464	0.0432
Total Pb	Total Zn	y = 7.9315 + 0.5468x	0.762	0.518	0.0168
Total Pb	Total Cu	y = 6.8933 + 2.5815x	0.587	0.344	0.0969
Total Ni	Total Pb	y = − 38.258 + 2.0441	0.500	0.244	0.1768
Available Pb	TOC	y = 7.6374 + 0.2498x	0.502	0.252	0.1677
Available Zn	Total Zn	y = 25.062 + 0.7926x	0.698	0.488	0.0364
Available Pb	Total Pb	y = 14.3814 + 0.955x	0.781	0.610	0.0130
Available Ni	Total Ni	y = − 44.81 + 20.017x	0.528	0.279	0.1438
NR	Total Pb	y = − 0.1285 + 0.668x	0.510	0.260	0.1605
NR	Available Cu	y = − 1.286 + 0.6395x	0.678	0.460	0.0445
PER	pH H_2_O	y = 4.1372–0.4371x	− 0.500	0.250	0.1705

**Figure 2:**
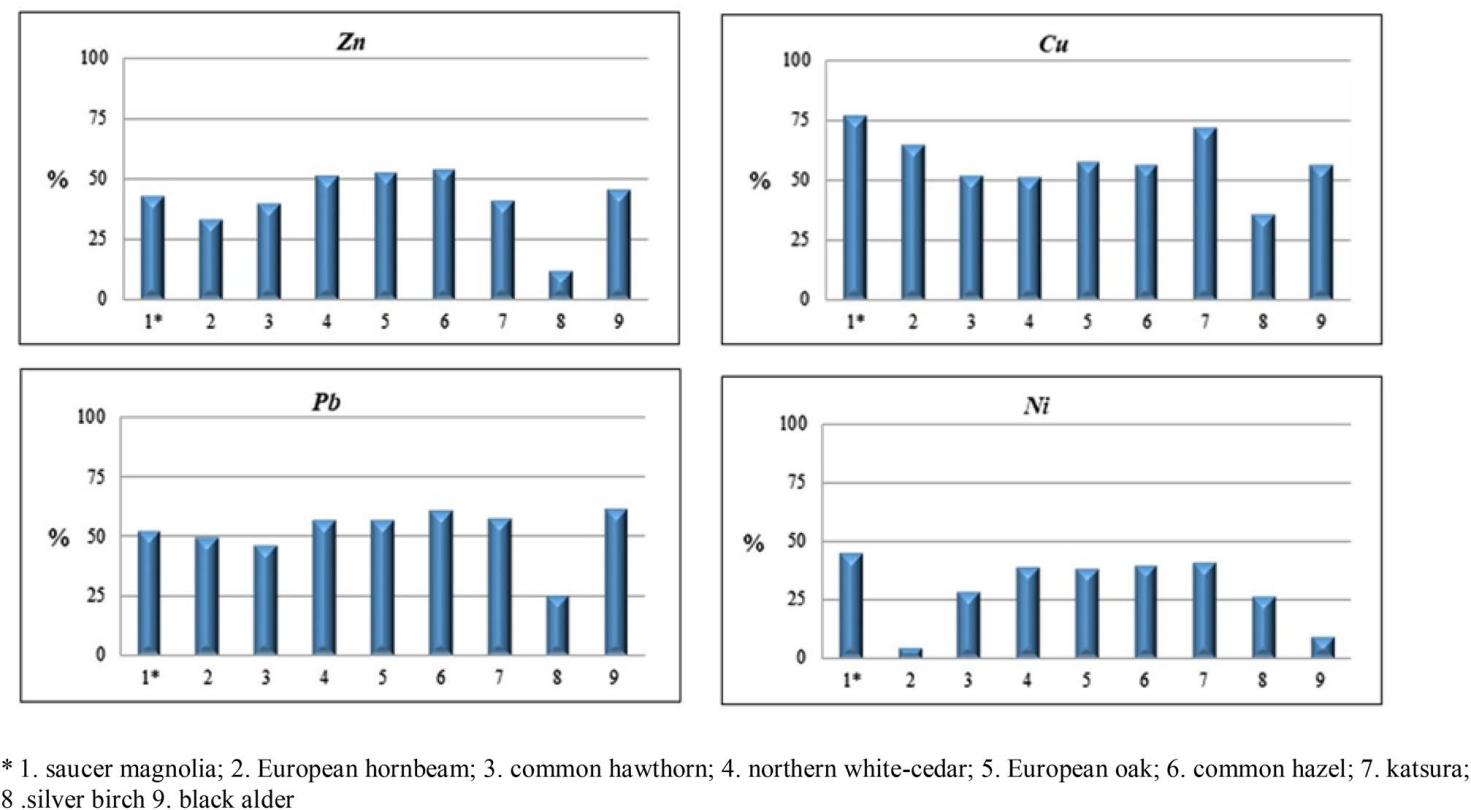
Availability factor (AF %) for some metals. Values followed by the same small letter are not significantly different at P < 0.05.

Heavy metals are taken up by plants mainly through the root system, but also through leaf surfaces. Metals are most easily taken up from the soil as free ions, while those in the form of complexes can be mobilised by active substances secreted by plant roots and then taken up^30^. Roots can release organic acids and metal chelators into the soil, causing the heavy metals to be released from insoluble soil complexes. The amount of metals taken up by plants depends on metal types, their content in the soil, the forms in which they occur and the plant species^31^. Modifying these factors can significantly affect the mobilisation or immobilisation of metals. The analysis of variance confirmed that the studied tree habitats significantly influenced on the content of the bioavailable metals Zn, Cu, Pb and Ni (Table 2). The content of bioavailable forms of the discussed metals ranged from 4.68 to 27.12 mg kg^−1^ for zinc, from 3.60 to 5.64 mg kg^−1^ for copper, from 6.36 to 22.06 mg kg^−1^ for lead and from 2.41 to 4.44 mg kg^−1^ for nickel. The mobility of heavy metals in the soil environment is determined by the forms in which they occur and the mechanisms by which they bind to organic and inorganic soil components. The prevailing soil conditions (especially physical and chemical properties), which significantly affect the mobility of trace elements and their bioavailability to plants, are also very important. How heavy metals bind, and thus their bioavailability, depends on many soil properties such as: organic matter content; pH; sorption capacity; the forms in which cations occur; concentrations of macro- and micronutrients; oxidation–reduction potential; and the activity of microorganisms. All these factors concurrently determine the amount of heavy metals accumulated in biological material or immobilised in soil particles^32,33^. It should also be noted that heavy-metal bioavailability also depends on the plant species. Different plant species and varieties growing under the same conditions display different capacities to take up metals from the soil^34^. The variation in the uptake of heavy metals between plant species is particularly high under acidic soil reaction, as the solubility of most elements toxic to plants increases. In our own studies, however, there were no significant relationships between soil pH and the content of bioavailable forms of the analysed metals. However, a significant relationship was found between the content of bioavailable forms of lead and TOC (*r* = 0.502, *p* = 0.1677). Organic matter is the basic adsorbent of heavy metals under acidic conditions and thus limits their availability to plants^35,36^. Heavy metals are generally taken up by plants proportionally to their concentration in the soil^37^, as confirmed by the obtained highly significant correlation coefficients between the content of available and total forms (Table 3). The high bioavailability (*AF*) values calculated for habitats of different species compositions (of 53.78% for Zn, 76.82% for Cu, 60.81% for Pb and 44.72% for Ni) may pose a risk of accumulation of these metals in plants (Fig. 2). Increased mobility of toxic elements in soil increases their accumulation in plants, which are thus seriously threatened^38^.

### Enzymes activity and its relation to other soil parameters

**Figure 3:**
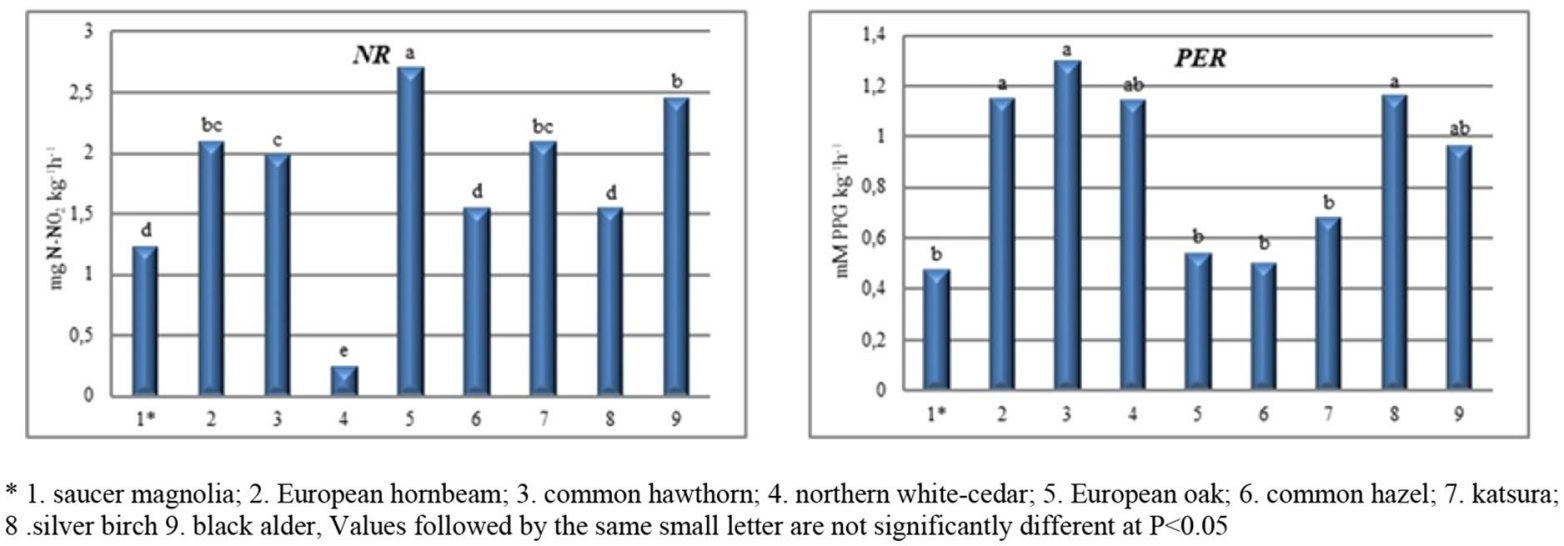
The activity of nitrate reductase (NR) and peroxidase (PER) in soil.

Based on the analysis of variance, it was found that tree species had a significant effect on the activity of nitrate reductase and peroxidases (Fig. 3). In terms of tree species, the significantly highest NR activity was found in soil under European oak (*Quercus robur* L.) (2.71 mg N-N0_2_ kg^-1^ h^−1^) and black alder (*Alnus glutinosa* Gaertn.) (2.46 mg N-N0_2_ kg^-1^ h^−1^). Measurement of the activity of nitrate reductase can be used as a diagnostic indicator for the availability of plant nitrogen^39^. NR activity depends primarily on the presence of nitrate, its substrate, in the soil. Thanks to its symbiosis with nitrogen-binding bacteria (*Schinzia alni*), black alder heavily enriches the soil with this element. The lowest NR activity was obtained in the soil within the zone of influence of northern white-cedar (0.250 mg N-N0_2_ kg^−1^ h^−1^), whose root system is shallow and flat with numerous small and dense roots growing close to the surface.

Single-factor analysis of variance showed a significant effect of tree species composition on peroxidase activity at the p = 0.05 level (Fig. 3). Much the highest PER activity was obtained in soil sampled from beneath common hawthorn (1.29 mM PPG kg^−1^ h^−1^) and silver birch (1.16 mM PPG kg^−1^ h^−1^). Birch is widely considered to positively affect many soil properties related to the fertility of a location. By contrast, the lowest activity was found in soil samples from under saucer magnolia (0.48 mM PPG kg^−1^ h^−1^). Peroxidases are enzymes that use H_2_O_2_ as an electron acceptor. These enzymes mediate in the key processes of a soil ecosystem, e.g. the degradation of lignin, humification and carbon mineralisation, by determining dissolved organic carbon content^40^. In the soil, these enzymes are mainly produced by fungi. Research by Mohsenzadeh et al.^41^ has shown that fungal peroxidases are capable of biodegrading petroleum derivative substances. According to Baldrian^42^, the species composition of a stand determines the spatial distribution and quantity of soil enzymes. Organic matter, which contains various components such as soluble sugars, organic acids and amino acids, as well as starch, cellulose and lignin, is delivered to the soil by root excretions and litter^14^. Because organic matter accumulates mainly in the upper layers, and our soil samples were collected from surface mineral levels (from 0 to 30 cm depth), we suggest that these species-specific differences in enzymatic activity also result from the difference in quality and quantity of above-ground litter. By contrast, Kotroczo et al.^43^ believe that plants cause greater changes in biochemical activity via their roots and root secretions than via the above-ground fall of detritus. Studies by Błońska^44^ and Zheng et al.^45^ have shown that enzymatic activity of soils varies by forest type. Soils from mixed forests were found to have higher contents of total and available nitrogen, phosphorus, potassium and organic carbon. Mixed forests also have about 94.9% greater abundance of soil microorganisms than pure-stand forests^46^.

Correlation analysis showed a significant relationship between total Pb content and NR activity (r = 0.510, p = 0.1605) (Table 3). The coefficient of determination (*R*^*2*^) showed that only 26% of the variability of nitrate reductase activity is due to the variability of Pbtot. The linear regression equation shows that increasing Pbtot content by 1 mg kg^−1^ NR increased activity by 0.668 mg N-N0_2_ kg^−1^ h^−1^. Nitrate reductase activity also correlated positively with bioavailable Cu content (r = 0.678, p = 0.0445). It should be emphasised that the present studies found no heavy-metal contents to be in excess of permissible levels. This indicates their natural accumulation in the soil, which did not inhibit the tested enzymes. This is probably related to the favourable physicochemical properties of the studied soils having reduced the mobility of heavy metals, at the same time reducing their toxicity to enzyme proteins. TOC content was not found to significantly correlate with nitrate reductase and peroxidases activity. The total lack of correlation between TOC content and the activity of the studied enzymes in the soil may be related to the low proportion of humic substances in the soil’s total organic matter content^47^. This limits the availability of easily absorbable carbon, which affects the development of microflora that produce soil enzymes.

### Statistical analysis

**Figure 4:**
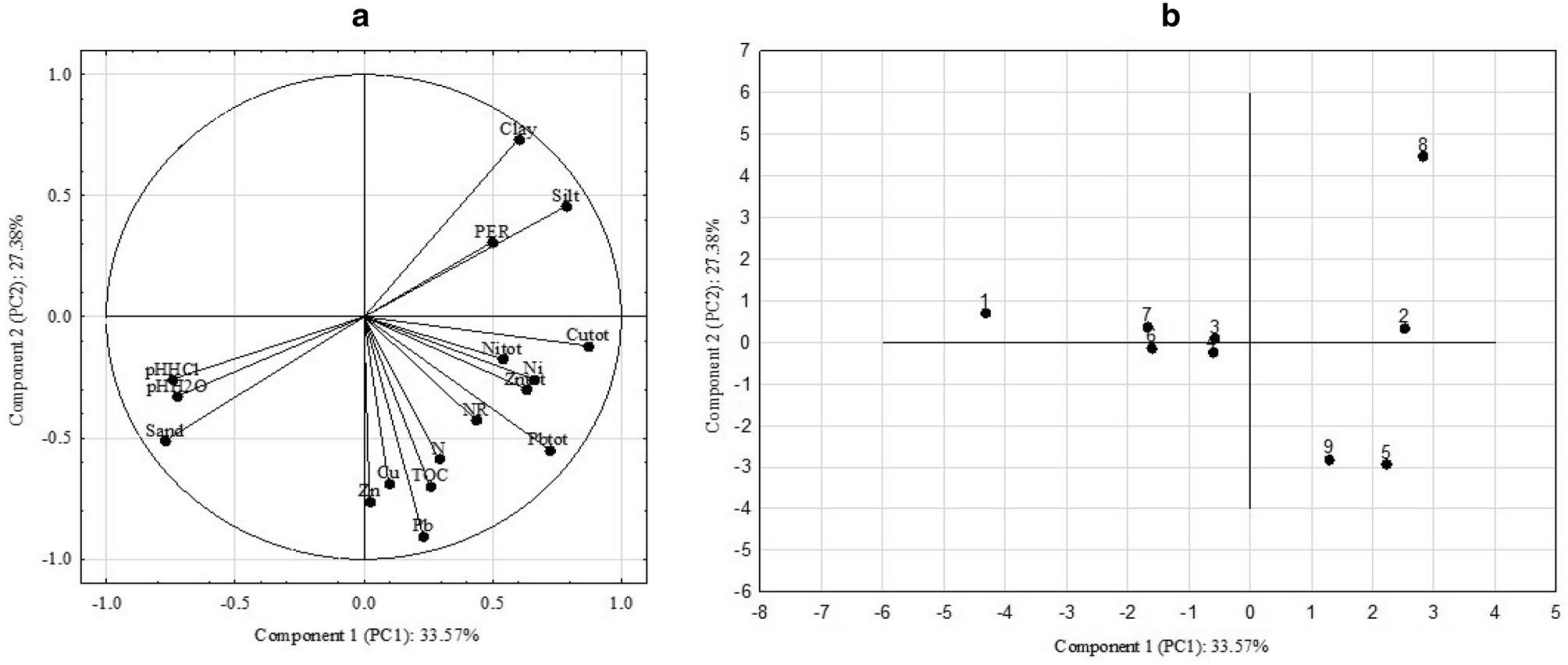
**(a, b)** Configuration of variables in the system of the first two axes PC1 and PC2 of principal components.

Principal component analysis (*PCA*) made it possible to detect general patterns in the relations between variables, and to describe and classify the studied parameters defined by the variables. On this basis, two principal components were identified (*PC1* and *PC2*) that explained 60.95% of the total change in variance (Table 4). Component 1 (*PC1*) was responsible for 33.57% of all variable component elements and correlated strongly with values for active acidity (− 0.728), exchangeable acidity (− 0.746), sand fraction (− 0.771) and silt fraction (0.783), as well as with total content of zinc (0.631), copper (0.874) and lead (0.720), and the content of bioavailable nickel forms (0.658). Component 2 (PC2), which explained 27.38% of overall variance, was related to clay fraction (0.734), TOC content (− 0.697), N (− 0.586), and the contents of bioavailable zinc (− 0.761), bioavailable copper (− 0.686) and bioavailable lead (− 0.904). From the graph (Fig. 4a) it can be concluded that the vectors of variables representing total contents of zinc, copper and lead—and bioavailable nickel—have the largest positive linear dependencies, allowing them to be grouped in with the first principal component. The longer the vector inside the circle, the more it is interdependent with the component. The decomposition of single variables’ effects on the contents of available forms of lead and zinc, and on clay fraction content, revealed that they had the dominant influence in determining the second component. On analysis of the distribution of the soil’s basic properties and the quantity of metals extracted from it in the coordinate factor graph, it can be concluded that the bioavailable forms of lead and copper correlated most strongly with organic matter content. The Fig. 4a also shows that total zinc and copper contents and peroxidase activity were negatively correlated with active and exchangeable acidity. Typically, peroxidases show maximum activity at pH = 5.0, which decreases as pH rises^48^. Soil pH controls enzyme activity by affecting its conformation and the adsorption of colloids^49^. Comparing sites from1 to 6 in Fig. 4b with the principal component forms and factor loadings, it can be concluded that soils were collected from the site 5 and 9 were characterized by the highest value of TOC, N, Pbtot, Nitot and content of bioavailable Pb and Ni and the activity of nitrate reductase. The next group includes sites 2 and 8 a high activity of peroxidase and the content of silt and clay.

**Table 4:** Values of the two extracted factor loadings for 17 elements.

Elements	Component matrix	
	PC1	PC2
Sand	− **0.771**	− 0.511
Silt	**0.783**	0.456
Clay	0.599	**0.734**
pH H_2_O	− **0.728**	− 0.329
pH KCl	− **0.746**	− 0.258
N	0.292	− **0.586**
TOC	0.257	− **0.697**
Total Zn	**0.631**	− 0.299
Total Cu	**0.874**	− 0.119
Total Pb	**0.720**	− 0.547
Total Ni	0.538	− 0.173
Available Zn	0.022	− **0.761**
Available Cu	0.096	− **0.686**
Available Pb	0.231	− **0.904**
Available Ni	**0.658**	− 0.257
NR	0.435	− 0.425
PER	0.498	0.309
Variation (%)	33.57	27.38
Eigenvalue	5.707	4.654

Bold value are statistically significant.

## Conclusion

The conducted analysis of variance confirmed the significant impact of the studied tree habitats on total content and bioavailable forms of metals. In the study area, permissible levels of Zn, Cu, Pb and Ni contents were not exceeded. It was found that high availability factor (AF) values calculated for species-specific habitats may represent a risk of accumulation of the tested metals in plants. It was also found that the activity of the studied oxidoreductive enzymes varied depending on the species composition of trees in the forest park. The correlation analysis carried out showed a significant negative effect of exchangeable acidity in determining total zinc and copper contents. Significant positive dependences were also found between bioavailable forms of Zn and clay fraction, as well as between bioavailable forms of Pb and TOC. Based on the principal component analysis (PCA), two principal components were identified that explained 60.95% of the total change in variance. The accumulation of heavy metals in soil is a slow process requiring research to be conducted systematically and data on the degree of soil contamination be updated. The conducted research brings new insights into changes in the content of heavy metals and enzymatic activity within the zone of influence of various tree species. Work in this field should be continued, because it will allow evaluate the ecological impacts of the gradual accumulation of potential contaminants in soils and plants to be assessed. Observing ongoing changes and forecasting their effects requires the use of specialised research and analysis techniques.
